# Decoding Cardiovascular Health: Carotid Intima-Media Thickness and Its Association With Coronary Artery Disease in the Indian Population

**DOI:** 10.7759/cureus.55836

**Published:** 2024-03-09

**Authors:** Aadithiyan Sekar, Aashika Parveen Amir, Abdul Majith Seeni Mohamed, Paarthipan Natarajan

**Affiliations:** 1 Department of Radiology, Saveetha Medical College and Hospital, Saveetha Institute of Medical and Technical Sciences, Saveetha University, Chennai, IND

**Keywords:** case-control study., biomarkers, indian population, cardiovascular risk factors, carotid intima-media thickness, coronary artery disease

## Abstract

Background

Coronary artery disease, as well as other cardiovascular diseases, poses a significant health burden globally. Understanding the relationship between clinical variables and coronary artery disease is crucial for effective management. This study explores the link between carotid intima-media thickness and different risk factors in the Indian population.

Aims and objectives

The primary objective of this study is to investigate the correlation between coronary artery disease and carotid intima-media thickness in a cohort of Indian individuals. Secondary objectives include analyzing the impact of demographic factors, lifestyle choices, and biomarkers on coronary artery disease risk.

Methodology

This study adopts an analytic, prospective case-control design spanning 18 months from July 2022 to December 2023. The research is conducted in a hospital setting, utilizing data from patients undergoing coronary angiography. The case group comprises 42 patients diagnosed with coronary artery disease, while the control group consists of 18 age-matched individuals without coronary artery disease. Demographic details, lifestyle factors, and biomarker levels are assessed. Statistical analyses involve Fisher’s exact tests, chi-square tests, ANOVA, and independent sample t-tests. Primary outcome measures include the association between carotid intima-media thickness and coronary artery disease, as well as the impact of demographic and lifestyle factors on coronary artery disease risk. Secondary outcome measures involve the predictive capability of carotid intima-media thickness through receiver operating characteristic (ROC) curve analysis.

Results

Significant findings include a notable association between gender and coronary artery disease, with a statistically significant relationship observed for smoking, alcohol consumption, and hypertension. Biomarkers such as high-sensitivity C-reactive protein (hs-CRP) and carotid intima-media thickness exhibit highly significant differences in coronary artery disease patients compared to controls.

Conclusions

This study underscores the importance of carotid intima-media thickness as a potential predictor for coronary artery disease in the Indian population. Gender, lifestyle choices, and certain biomarkers significantly influence coronary artery disease risk. These findings contribute to a nuanced understanding of coronary artery disease etiology and risk stratification. This study sheds light on the intricate interplay of clinical factors influencing coronary artery disease in the Indian population, paving the way for enhanced risk assessment and preventative strategies.

## Introduction

Coronary artery disease (CAD) stands as a pervasive global health issue, accounting for a substantial burden of morbidity and mortality. The past 25 years have witnessed a significant surge in CAD cases in India [1], necessitating a critical re-evaluation of diagnostic methodologies to address the evolving landscape of cardiovascular health. CAD encompasses a spectrum of cardiovascular conditions, like unstable angina, stable angina, myocardial infarction (MI), and sudden cardiac death, all rooted in the intricate interplay between oxygen demand and supply to the myocardium.

Catheter coronary angiography (CAG) is the primary method for diagnosing coronary artery conditions, which plays a pivotal role in understanding CAD [2]. However, their invasive nature and limitations in visualizing coronary artery plaques underscore the need for innovative, non-invasive diagnostic tools. In this context, our study endeavors to explore the potential of carotid intima-media thickness (CIMT) measurement, utilizing carotid Doppler ultrasonography (USG), as a promising avenue for non-invasive CAD diagnosis.

Our main aim is to calculate the efficacy of CIMT measurement as the screening method to detect CAD early. Through meticulous assessment of CIMT using carotid Doppler USG, we aim to establish its reliability and cost-effectiveness as a diagnostic tool.

Furthermore, we seek to study the interrelation between CIMT values and the conditions of CAD. By comparing CIMT measurements in individuals with and without CAD, our study aspires to shed light on its potential role as a prognostic indicator, offering valuable insights for risk stratification in clinical settings. Furthermore, the study investigates the role of high-sensitivity C-reactive protein (hs-CRP), an inflammatory biomarker, in predicting cardiovascular risk.

With CAD emerging as a leading cause of global mortality [3], the significance of our study lies in its potential to redefine early diagnosis and risk assessment paradigms. If CIMT proves to be a robust screening tool and exhibits a correlation with the severity of CAD, it could herald a transformative shift in preventive strategies and individualized patient care.

## Materials and methods

In a novel approach to evaluate the correlation between CIMT and CAD, a prospective, analytical case-control study was conducted involving 60 patients of diverse age groups and genders. These individuals, undergoing CAG due to suspected CAD at a tertiary care center, were subsequently referred for CIMT measurement by USG, with the procedure's findings kept confidential from the radiologists to maintain the study's integrity. The methodology involved high-resolution B-mode USG imaging of the carotid arteries, where precise measurements of the intima-media thickness were taken from predetermined segments of the common carotid artery, emphasizing the importance of diastolic phase imaging for accurate assessment.

Following the comprehensive diagnostic assessments through both CAG and CIMT, participants were categorized into two groups based on the CAG outcomes. Group I consisted of individuals with no significant coronary lesions, effectively acting as the control group to investigate the potential link between increased CIMT values and the presence of CAD. Group II included patients diagnosed with at least one significant lesion (over 50% obstruction) in the primary coronary artery branches. This group was further segmented according to the angiographically determined number of affected vessels, aiming to delve deeper into the relationship between CIMT measurements and the extent of CAD.

The study's analysis phase was meticulously designed to compare the findings within Group II to either confirm or challenge the hypothesis that CIMT measurements can reliably predict the severity of CAD. Statistical methods employed included percentage and frequency analysis for continuous variables, with mean and standard deviation calculations for categorical data. The chi-square test was utilized to assess the significance of qualitative categorical data, ensuring a robust statistical foundation for the study's conclusions.

A critical component of the study's analytical framework was the use of the receiver operating characteristic (ROC) curve, focusing on evaluating the sensitivity and specificity of CIMT as a predictive tool for CAD. This statistical analysis aimed to establish a definitive correlation between CIMT values and CAD presence or severity, with a significant p-value (less than 0.05) indicating a strong predictive capability.

The findings of this comprehensive study have the potential to significantly impact clinical practices by validating the use of CIMT, a non-invasive and accessible diagnostic measure, as an effective tool for predicting the presence and extent of CAD. Such insights could lead to earlier intervention and more personalized treatment strategies for patients at risk of or suffering from CAD, ultimately improving clinical outcomes in this patient population.

## Results

Among 60 patients considered for the study, with 35 males and 25 females (Table 1), 42 were diagnosed with CAD, comprising 28 males and 14 females, while 18 patients, consisting of seven males and 11 females, were found to be free of CAD. According to the age distribution, 25.0% of patients were in age bracket of 41 and 50, 31.7% were from 51 and 60 years of age, and 43.3% were older than 60 (Table 2).

**Table 1:** Gender distribution

Gender	Frequency	Percentage
Female	25	41.7
Male	35	58.3
Total	60	100.0

**Table 2:** Age distribution

Age	Frequency	Percentage
41-50 years	15	25.0
51-60 years	19	31.7
Above 60 years	26	43.3
Total	60	100.0

**Figure 1:**
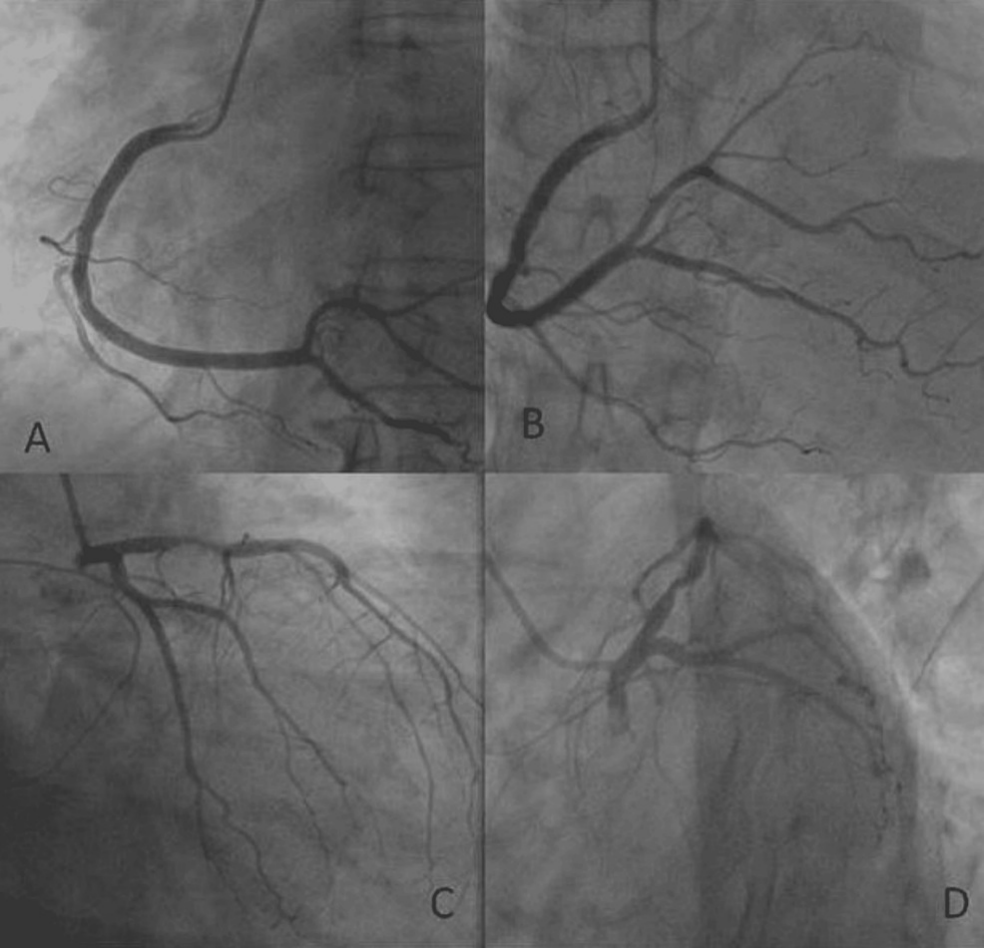
(Clockwise) Left anterior oblique view and left anterior oblique cranial view for the right coronary artery show no evidence of stenosis in the right coronary artery and its branches, right anterior oblique caudal and left anterior oblique caudal/spider views for the left coronary artery and its branches shows >80% stenosis in the proximal left anterior descending artery.

**Figure 2:**
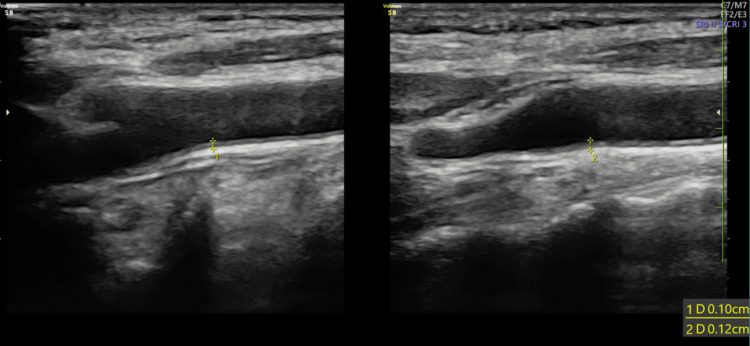
B-mode ultrasonography of bilateral common carotid artery (longitudinal view) shows carotid intima-media thickness of 1.0 and 1.2 mm in the far wall of right and left common carotid artery, respectively.

A male in his 40s with angina and a positive treadmill test came to the hospital. A catheter coronary angiogram was done, which resulted in 80% stenosis in the left anterior descending artery. Correspondingly, B-mode USG showed a CIMT of 1.0 and 1.2 mm, respectively (Figures 1-2). 

A Pearson’s chi-square test was implemented to test the relationship between age and diagnosis, yielding a χ² value of 5.207 with a p-value of 0.074. The result indicates no statistical significance between age and the diagnosis of CAD.

Regarding gender distribution, 41.7% were females, and 58.3% were males. Pearson’s chi-square test for gender and diagnosis produced a χ² value of 4.000 with a p-value of 0.046, demonstrating statistical significance between gender and the diagnosis of CAD (Table 3).

**Table 3:** Comparison of gender, hypertension, smoking, and alcohol with diagnosis CAD, coronary artery disease; HTN, hypertension **Highly statistically significant at p < 0.01 *Significant at p < 0.05

Gender	Count and percentage	Diagnosis		Total	χ² value	p-value
		CAD	Non-CAD			
Female	Count	14	11	25	4.000	0.046*
	%	33.3%	61.1%	41.7%		
Male	Count	28	7	35		
	%	66.7%	38.9%	58.3%		
Total	Count	42	18	60		
	%	100.0%	100.0%	100.0%		
HTN	Count and percentage	Diagnosis		Total	χ² value	p-value
		CAD	Non-CAD			
Absent	Count	17	14	31	7.020	0.01*
	%	40.5%	77.8%	51.7%		
Present	Count	25	4	29		
	%	59.5%	22.2%	48.3%		
Total	Count	42	18	60		
	%	100.0%	100.0%	100.0%		
Smoking	Count and percentage	Diagnosis		Total	χ² value	p-value
		CAD	Non-CAD			
Absent	Count	25	16	41	5.021	0.034*
	%	59.5%	88.9%	68.3%		
Present	Count	17	2	19		
	%	40.5%	11.1%	31.7%		
Total	Count	42	18	60		
	%	100.0%	100.0%	100.0%		
Alcohol	Count and percentage	Diagnosis		Total	χ² value	p-value
		CAD	Non-CAD			
Absent	Count	17	15	32	9.298	0.004**
	%	40.5%	83.3%	53.3%		
Present	Count	25	3	28		
	%	59.5%	16.7%	46.7%		
Total	Count	42	18	60		
	%	100.0%	100.0%	100.0%		

In the comparison of comorbidities with CAD diagnosis, Fisher’s exact tests were applied. Diabetes mellitus (DM) showed no statistical significance (χ² = 2.310, p = 0.155 > 0.05), while hypertension (HTN) exhibited statistical significance (χ² = 7.020, p = 0.011 < 0.05) with the diagnosis of CAD (Table 3). Old ischemic heart disease (Old IHD) did not show statistical significance (χ² = 3.956, p = 0.091 > 0.05).

Smoking demonstrated statistical significance with CAD diagnosis (χ² = 5.021, p = 0.034 < 0.05) (Table 3), and alcohol consumption showed high statistical significance (χ² = 9.298, p = 0.004 < 0.01) (Table 3). Diet, however, did not exhibit statistical significance with CAD diagnosis (χ² = 0.000, p = 1.0 > 0.05).

Biomarkers, such as CIMT and hs-CRP, were evaluated. The independent sample t-tests for hs-CRP (Table 4) and CIMT (Table 5) showed high statistical significance differences at p < 0.01 level (t-values = 5.581 and 8.739, respectively). 

**Table 4:** Comparison of high-sensitivity C-reactive protein (hs-CRP) with diagnosis by independent sample t-test CAD, coronary artery disease **Highly statistically significant at p < 0.01 level

Biological marker	Diagnosis	N	Mean	SD	t-value	p-value
hs-CRP	CAD	42	3.6	1.1	5.581	0.0005**
	Non-CAD	18	2.1	0.4		

**Table 5:** Comparison of carotid intima-media thickness (CIMT) with diagnosis by independent sample t-test CAD, coronary artery disease **Highly statistically significant at p < 0.01 level

Parameter considered	Diagnosis	N	Mean	SD	t-value	p-value
CIMT	CAD	42	1.4	0.3	8.739	0.0005**
	Non-CAD	18	0.8	0.1		

Furthermore, CIMT association with the number of vessels involved in CAD was examined using one-way ANOVA, revealing high statistical significance (F-value = 15.231; p < 0.01) (Table 6). Post hoc tests demonstrated higher statistical significance at p < 0.01 and statistical significance at p < 0.05 levels (Table 7).

**Table 6:** Comparison of carotid intima-media thickness with number of vessels in coronary artery disease **Highly statistically significant at p < 0.01 level

Vessels	N	Mean	SD	F-value	p-value
Single	18	1.24	0.20	15.231	0.0005**
Double	16	1.46	0.24		
Triple	8	1.76	0.24		

**Table 7:** Comparison of carotid intima-media thickness with number of vessels in coronary artery disease by one-way ANOVA test CI, confidence interval; LB, lower bound; MD, mean deviation between the compared entities; UB, upper bound **Highly statistically significant at p < 0.01 *Significant at p < 0.05

Number of vessels involved		MD	Standard error	p-value	95% CI	
					LB	UB
Single	Double	-0.22299^*^	0.077	0.017*	-0.411	-0.035
	Triple	-0.51986^*^	0.095	0.0005**	-0.752	-0.287
Double	Triple	-0.29688^*^	0.097	0.011*	-0.534	-0.060

**Figure 3:**
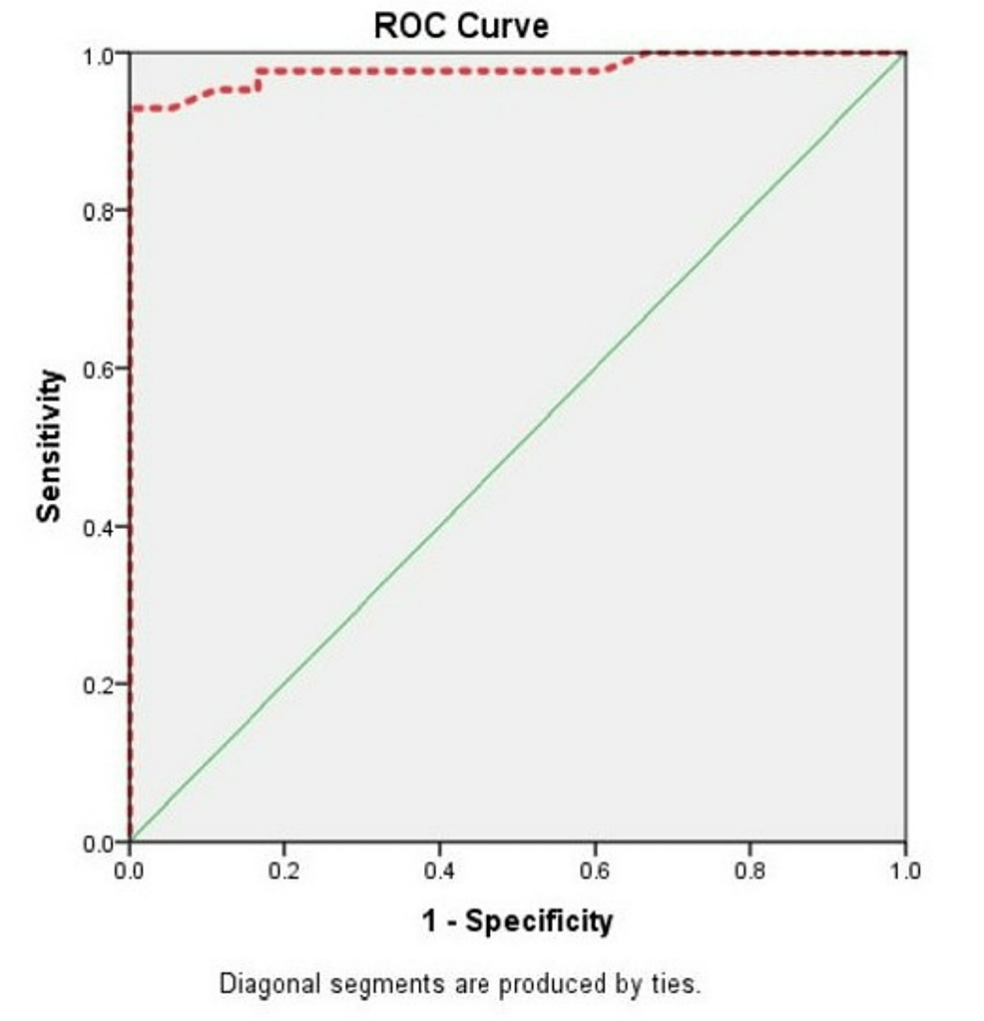
Receiver operating characteristic (ROC) curve analysis for carotid intima-media thickness with coronary artery disease diagnosis yielded an area under the curve of 0.979, with a p-value of 0.0005 < 0.01.

ROC analysis for CIMT with CAD diagnosis resulted in an area under the curve of 0.9 (Figure 3), with p-value = 0.0005 < 0.01 (Table 8). The sensitivity, specificity, positive predictive value, negative predictive value, and accuracy were 92.9%, 94.4%, 97.5%, 85.0%, and 93.3%, respectively (Table 9).

**Table 8:** Comparison of carotid intima-media thickness with diagnosis using receiver operating characteristic (ROC) curve CI, confidence interval; LB, lower bound; UB, upper bound **Highly statistically significant at p < 0.01 level

Area	p-value	95% CI	
		LB	UB
0.979	0.0005**	0.946	1.000

**Table 9:** The sensitivity, specificity, positive predictive value, negative predictive value, and accuracy

CIMT	Diagnosis		Total	Parameter	Value
				Sensitivity	92.9
	CAD	Non-CAD		Specificity	94.4
≥0.95	39	1	40	PPV	97.5
<0.95	3	17	20	NPV	85.0
Total	42	18	60	Accuracy	93.3

In summary, this comprehensive analysis underscores the significance of gender, HTN, smoking, alcohol consumption, hs-CRP, and CIMT as potential indicators of CAD, providing valuable insights for risk stratification and early diagnosis.

## Discussion

In clinical practice, both interventional and non-interventional methods play pivotal roles in detecting atherosclerosis. Measurement of CIMT, endorsed by the American Heart Association, stands out as a valuable non-invasive method, particularly when assessed through B-mode USG [4]. Age and sex both affect normal intima-media thickness (IMT) levels and reference ranges; all carotid segments show a considerable continuous increase in IMT with age, and men have significantly higher IMT values than women [5-7]. It is debatable which IMT levels should be regarded as abnormal. It would be erroneous to divide the link between IMT and cardiovascular risk into two categories by setting a threshold IMT value. However, it should be emphasized that CIMT > 0.9 mm was confirmed as the marker of silent organ damage in the most recent European Society of Hypertension (ESH)/European Society of Cardiology (ESC) HTN recommendations (2013), even though elderly and middle-aged patients had higher threshold values, which show significant cardiovascular risk [8,9].

The early link between CIMT and subsequent coronary events was established in the Kuopio Ischemic Heart Disease Risk Factor study, where a 0.1 mm rise in IMT correlated with an 11% higher risk of MI during follow-up [10]. Our study, conducted at a tertiary care center, included 60 patients with cardiac symptoms, either diagnosed with or without CAD. Utilizing B-mode carotid artery USG, we effectively assessed early atherosclerotic changes. Age, sex, CAD, and hs-CRP were identified as factors contributing to the higher value of intima-media complex thickness. Most of the patients in this study were above 60 years of age, which is consistent with the age-related prevalence of CAD. Males were more affected than females, with the highest incidence in the 50-60 years age group. In our control group with normal CAG, no contrast-related complications occurred. Catheter CAG served as the primary method for diagnosing and predicting the extent of CAD (single, double, or triple/multiple vessel involvement).

The statistical analysis resulted in no significant correlation between age and CAD diagnosis, while gender exhibited statistical significance. Comorbidities, such as DM, HTN, Old IHD, smoking, and alcohol consumption were assessed. HTN demonstrated statistical significance with CAD diagnosis, while smoking and alcohol consumption exhibited highly significant correlations. Age, gender, DM, Old IHD, and diet did not show statistical significance with CAD diagnosis. The study revealed a notable association between hs-CRP levels and CAD diagnosis, emphasizing the role of inflammation in atherosclerosis. The mean hs-CRP in CAD patients was significantly higher compared to non-CAD patients. Comparing our results with previous studies, our findings align with the positive association between elevated CIMT and CAD, supporting the utility of CIMT in assessing the severity of the condition. Other studies have corroborated the predictive value of CIMT in identifying individuals at high cardiovascular risk.

Longitudinal studies, such as Chan et al.'s monitoring of 152 CAD patients for six to 11 months and Bots et al.'s Rotterdam trial, reinforce the significance of CIMT as a non-interventional marker for vascular events and MI prediction [11,12]. According to Demircan et al., compared to individuals with stable angina pectoris, those with acute coronary syndrome had significantly greater CIMTs [13]. A separate study showed that the ability to predict angiographic CAD was 85.7% sensitive and 85.1% specific at a maximal CIMT value of 0.956 mm [14]. According to the research by Kablak-Ziembicka et al., higher CIMT was linearly and positively connected to CAD; participants who had more vessels involved in their conditions had bigger increases in CIMT [15]. Additionally, according to their research, a CIMT of more than 1.15 mm was associated with a 94% risk of developing CAD.

Additionally, a strong positive linear trend between CIMT and the total number of vessels involved was observed by Geroulakos et al. [16]. If a CIMT > 0.85mm, correlated for age, is considered as the cut-off point for the prediction of CAD, the study also showed a strong specificity and positive predictive value for the incidence of CAD [17]. The Edinburgh Artery Study established a correlation between increased common carotid artery (CCA)-IMT and cardiovascular events [18]. The Framingham Offspring and Charlottesville studies reinforced associations between CIMT and cardiovascular outcomes, advocating CIMT as a potential marker for risk assessment [19,20]. In Firefighters and Their Endothelium (FATE), 1,574 participants aged 49.4 (9.9) years showed an HR of 1.45 (95% CI: 1.15-1.83) for CV events [21]. Osaka Follow-up Study for Carotid Atherosclerosis 2 (OSACA2) found a relative risk (RR) of 1.57 (95% CI: 1.11-2.20) for cardiovascular outcomes [22]. Tromsø Study indicated elevated IMT quartiles associated with MI [23].

A smaller study population and a shorter duration of follow-up of cases were the two limitations of the study. Our investigation contributes to the growing body of evidence supporting CIMT as a valuable tool for early atherosclerosis detection and risk stratification. The integration of non-invasive methods like CIMT measurement into routine clinical practice holds promise for enhancing cardiovascular risk assessment and optimizing preventive strategies.

## Conclusions

The overall accuracy of CIMT thickness measurement as a screening technique in finding CAD is 93.3%. From this study, we concluded that CIMT measurement using carotid Doppler USG can be used as a reliable screening technique for early diagnosis of CAD. In addition, values of CIMT can be used to establish the extent of CAD. The hs-CRP value also increases with the increase in the severity of the disease. There is a positive correlation between DM and the IMT and also with the history of smoking and CIMT thickness.
